# An ancient schwannoma with venous malformation-like features of the tongue: A case report and review of the literature

**DOI:** 10.1097/MD.0000000000042936

**Published:** 2025-06-20

**Authors:** Tatsufumi Fujimoto, Kana Hasegawa, Wataru Kumamaru, Toru Chikui, Yuta Yanai, Minami Shibuya, Shinsuke Fujii, Kazunori Yoshiura, Shintaro Kawano, Tamotsu Kiyoshima

**Affiliations:** a Laboratory of Oral Pathology, Division of Maxillofacial Diagnostic and Surgical Sciences, Faculty of Dental Science, Kyushu University, Fukuoka, Japan; b Sections of Oral and Maxillofacial Surgery, Division of Maxillofacial Diagnostic and Surgical Sciences, Faculty of Dental Science, Kyushu University, Fukuoka, Japan; c Oral and Maxillofacial Radiology, Division of Maxillofacial Diagnostic and Surgical Sciences, Faculty of Dental Science, Kyushu University, Fukuoka, Japan; d Dento-craniofacial Development and Regeneration Research Center, Faculty of Dental Science, Kyushu University, Fukuoka, Japan; e Maxillofacial Oncology, Division of Maxillofacial Diagnostic and Surgical Sciences, Faculty of Dental Science, Kyushu University, Fukuoka, Japan.

**Keywords:** ancient schwannoma, composite lesion, tongue, venous malformation-like feature

## Abstract

**Rationale:**

Composite lesions of neurogenic tumors with vascular malformations, which were once included among hemangiomas, are extremely rare and can be classified into 2 types: conjoined and discrete associations, with the former representing a single lesion of vascular malformation within tumor tissue. To date, we have found 24 composite lesions (conjoined association type) of schwannomas coexisting with vascular malformations. However, such composite lesions should be interpreted with caution.

**Patient concerns:**

A 29-year-old woman had recognized swelling of the tongue 5 years prior to her initial hospital visit and the lesion had recently been slowly growing.

**Diagnoses:**

Based on clinical examinations, including imaging studies, the tumorous lesion was clinically diagnosed as a schwannoma, but a benign salivary gland tumor and venous malformation (VM) were not completely ruled out.

**Interventions:**

The lesion was excised with safety margin.

**Outcomes:**

The excised sample revealed 2 intriguing features; however, the lesion was diagnosed as an ancient schwannoma with VM-like features because of the lack of definitive findings that would make these lesions a composite of independent lesions. There was no evidence of recurrence or distant metastasis at the 16-month follow-up after excision.

**Lessons:**

Ancient schwannomas of the oral cavity are rare. In particular, schwannomas that appear as composite lesions of schwannomas with vascular malformations are extremely rare. Here, we report the first case of an ancient schwannoma with VM-like features of the tongue. It is important for clinicians to accumulate information on these rare cases to make an accurate preoperative diagnosis and to plan appropriate treatment.

## 1. Introduction

The coexistence of neurogenic tumors and hemangiomas, called composite tumors, is very rare.^[1–3]^ Based on their relationship, composite tumors can be classified into 2 types: conjoined and discrete associations. The former represents a single lesion of different tumors, and the latter is a type of different tumors occurred in distinct and separate locations.^[1–3]^ However, since the term hemangiomas is used to include vascular malformations that are not considered true vascular tumors, such as cavernous hemangiomas,^[4–6]^ the aforementioned composite tumors should be referred to as composite lesions herein. Composite lesions of schwannomas and venous malformations (VMs) are extremely rare entity.^[1–3]^ To our knowledge, 40 cases of composite lesions of neurogenic tumors, including schwannomas, neurofibromas, and gangliogliomas, and vascular malformations, have been reported in the literature. Additionally, 22 cases of conjoined associations were reported as composite lesions of schwannoma and VM (Table 1).^[1,3,7–16]^ According to previous reports, there are no cases of composite lesions in the oral cavity. The lack of substantial information regarding composite lesions makes preoperative diagnosis difficult. Furthermore, most clinicians find it difficult to clinically differentiate this type of composite lesion from other lesions because of its rarity, as in our previously reported cases.^[17–19]^ In addition, such composite lesions must be interpreted with caution. For example, there is not enough evidence to assume that each site is an independent lesion, sites suggestive of vascular malformations may be secondary changes, and that they are included in the histological findings of the schwannoma subtype because several vessels of various sizes are seen within conventional schwannomas.^[20]^

**Table 1:** Literature review: composite lesions (conjoined associations) of schwannoma and vascular malformation (hemangioma) from previous reports.

Case number	Age (year), sex	References	Reported year	Type of hemangioma	Size of tumor (cm)	Treatment	Location
1	17, M	Willis^[7]^	1967	Cavernous hemangioma	3	Resection	Mediastinum
2	77, M	Bojsen et al^[8]^	1978	Cavernous hemangioma	2 × 1.5 × 1	Resection	Left 4th lumbar root
3	57, F	Bojsen et al^[8]^	1978	Cavernous hemangioma	4 × 4 × 5	Resection	Right cerebellopontine angle
4	45, M	Bojsen et al^[8]^	1978	Cavernous hemangioma	2.5 × 1 × 1	Resection	Left 4th cervical root
5	36, M	Bojsen et al^[8]^	1978	Cavernous hemangioma	2.5 × 1.5 × 1.5	Resection	Sensory root of 3rd left lumbar nerve
6	25, F	Bojsen et al^[8]^	1978	Cavernous hemangioma	2 × 1.5 × 1	Resection	Medial cord of right branchial plexus
7	60, M	Kasantikul et al^[9]^	1979	Cavernous hemangioma	-	-	Ulnar nerve
8	58, F	Kasantikul et al^[9]^	1979	Cavernous hemangioma	-	-	Peroneal nerve
9	66, F	Kasantikul et al^[9]^	1979	Cavernous hemangioma	-	-	Spinal cord C1-C2
10	60, F	Kasantikul et al^[9]^	1979	Cavernous hemangioma	-	-	Cranial nerve Ⅷ
11	55, M	Kasantikul et al^[9]^	1979	Arterial hemangioma	-	-	Cranial nerve Ⅷ
12	40, F	Kasantikul et al^[9]^	1979	Cavernous hemangioma	-	-	Cranial nerve Ⅷ
13	64, M	Kasantikul et al^[9]^	1979	Cavernous hemangioma	-	-	Cranial nerve Ⅷ
14	47, M	Kasantikul et al^[9]^	1979	Cavernous hemangioma	-	-	Cranial nerve Ⅷ
15	77, F	Pasquier et al^[10]^	1980	Cavernous hemangioma	10 × 9 × 5	Resection	Mediastinum
16	31, F	Kasantikul et al^[11]^	1982	Cavernous hemangioma	-	Resection	Cranial nerve Ⅴ
17	54, M	Kasantikul et al^[12]^	1984	Cavernous hemangioma	6 × 5 × 5	Resection	Parapharyngeal
18	48, F	Kasantikul et al^[13]^	1987	Cavernous hemangioma	4 × 5	Resection	Parasellar
19	74, M	Asari et al^[14]^	1992	Cavernous hemangioma	3	Resection	Cranial nerve Ⅴ
20	74, M	Asari et al^[14]^	1992	Cavernous hemangioma	3	Resection	Cranial nerve Ⅴ
21	76, M	Feiz-Erfan et al^[3]^	2006	Cavernous hemangioma	-	Resection	Cranial nerve Ⅷ
22	34, M	Wilson et al^[15]^	2016	Arterial hemangioma	2.2 × 2.1 × 1.9	Resection	Left temporal lobe
23	30, M	Sethi et al^[16]^	2020	Cavernous hemangioma	5 × 3 × 2	Resection	Left Arm subcutaneous
24	24, F	Ramkumar^[1]^	2021	Cavernous hemangioma	6 × 4 × 4	Resection	Head subcutaneous

“M” and “F” in sex are male and female, respectively.

“-” means that the item is not mentioned in the corresponding reference.

Based on histopathological features, schwannomas are divided into several types, with classic or conventional schwannomas being the most common. Classic or conventional schwannomas consist of 2 patterns that are primarily hypercellular areas (so-called “Antoni A areas”) and hypocellular areas (so-called “Antoni B areas”). Other subtypes of schwannomas include ancient schwannoma, cellular schwannoma, plexiform schwannoma, epithelioid cell schwannoma, and microcystic reticular schwannoma.^[20–23]^ Ancient schwannomas are usually found in deep tissues, especially in the retroperitoneum, but are rare in the oral and maxillofacial regions. Histological features include degenerative-type nuclear atypia and stromal changes. Ancient changes include vascular wall and diffuse stromal hyalinization, mucous deposition, cyst formation, hemorrhage, calcification, and infiltration of siderophages/histiocytes intermingled with the neoplastic cells. Marked nuclear atypia is the most alarming feature of ancient schwannomas, and a pitfall in differentiating them from malignancies.^[20–23]^

Herein, we report the first case of an ancient schwannoma with VM-like features of the tongue. This is an extremely rare case due to the location of the lesion and its unique histopathological features.^[20–23]^ It is important for clinicians to accumulate information on these rare cases to make an accurate preoperative diagnosis and plan appropriate treatment.

## 2. Case report

**Figure 1.**
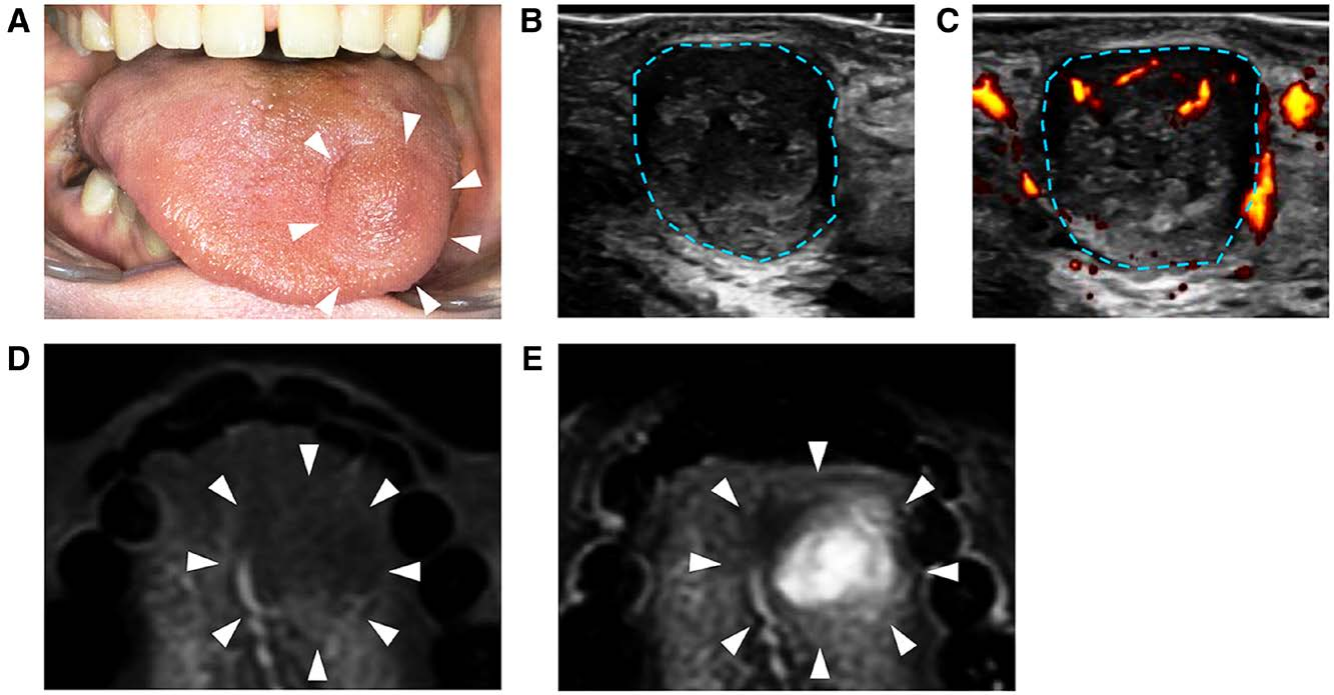
Macroscopic appearance, and US and MRI findings of the lesion. (A) Macroscopic image of the tongue at the initial examination. The soft-elastic, low-mobility, and well-defined mass was covered with normal-colored mucosa. Arrowheads indicate the mass location. (B) US showing an approximately 10 × 11 mm well-defined mass. The blue dotted line indicates the mass margin. (C) Doppler US showing abundant blood flow (yellow and red). The blue dotted line indicates the mass margin. (D) On T1-weighted MRI, the lesion was hypointense (arrowheads). (E) T2-weighted water-only MRI showing an area of heterogeneous and high-signal intensity (arrowheads). US = ultrasonography, MRI = magnetic resonance imaging.

A 29-year-old woman was referred to the Department of Oral and Maxillofacial Surgery at Kyushu University Hospital (Fukuoka, Japan) for an examination and treatment. She had aware of swelling of the tongue for 5 years prior to the initial visit. While she had gone untreated because there were no symptoms and no swelling, the mass had recently been slowly growing. The patient was otherwise healthy with no significant diseases. An initial oral examination revealed that the tumorous lesion was a 10 × 10 mm spherical, well-defined, soft-elastic, and low-mobility mass in the back of the tongue on the left side, approximately 20 mm from the apex of the tongue (Fig. 1A). The mucosa overlying the mass appeared normal and did not fade upon palpation. She had no notable clinical findings except for the above.

Ultrasonography (US) revealed an area of approximately 10 × 11 mm with a well-defined mass in the tongue. The interior of the lesion was heterogeneous, with posterior acoustic enhancement (Fig. 1B). Doppler US revealed abundant blood flow at the center and margin of the tumor (Fig. 1C). Magnetic resonance imaging (MRI) revealed a mass measuring approximately 8 × 10 × 8 mm in the tongue. On T1-weighted images, the intensity of the mass was low (Fig. 1D), whereas on T2-weighted water-only images, it was high (Fig. 1E). The apparent diffusion coefficient was 1.7 × 10^-3^ mm^2^/s and appeared high. The tumorous lesion was clinically diagnosed as a schwannoma, but benign salivary gland tumor and VM were not completely ruled out.

**Figure 2.**
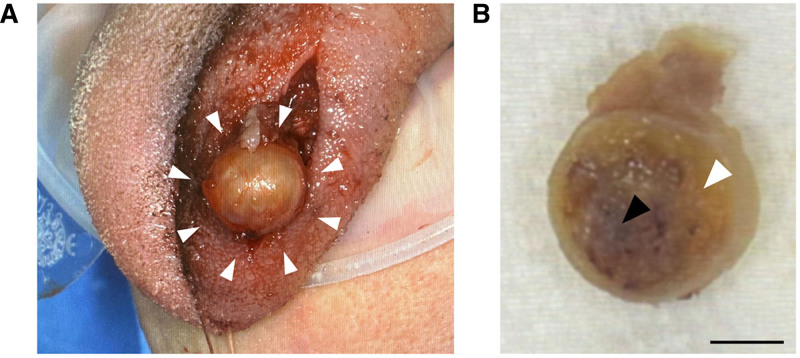
Intraoperative photo and cut surface image of the extirpated tumor mass. (A) The lesion was an encapsulated, well-circumscribed round mass in the tongue, and the surface was yellowish white. Arrowheads indicate the mass. (B) The cut surface of the tumor had a yellowish white lesion (white arrowhead) and reddish vascular-like lesion (black arrowhead). Scale bar, 10 mm.

The histological findings of the needle biopsy were suggestive of schwannomas; therefore, the tumor was removed with 1 layer of muscle. The extirpated sample was well circumscribed and measured 13 × 10 × 10 mm (Fig. 2A). The cut surface was yellowish white in the marginal regions and had vascular-like structures of varying sizes in the center. There was no apparent invasion of the tumor tissue into the surrounding soft tissue (Fig. 2B).

**Figure 3.**
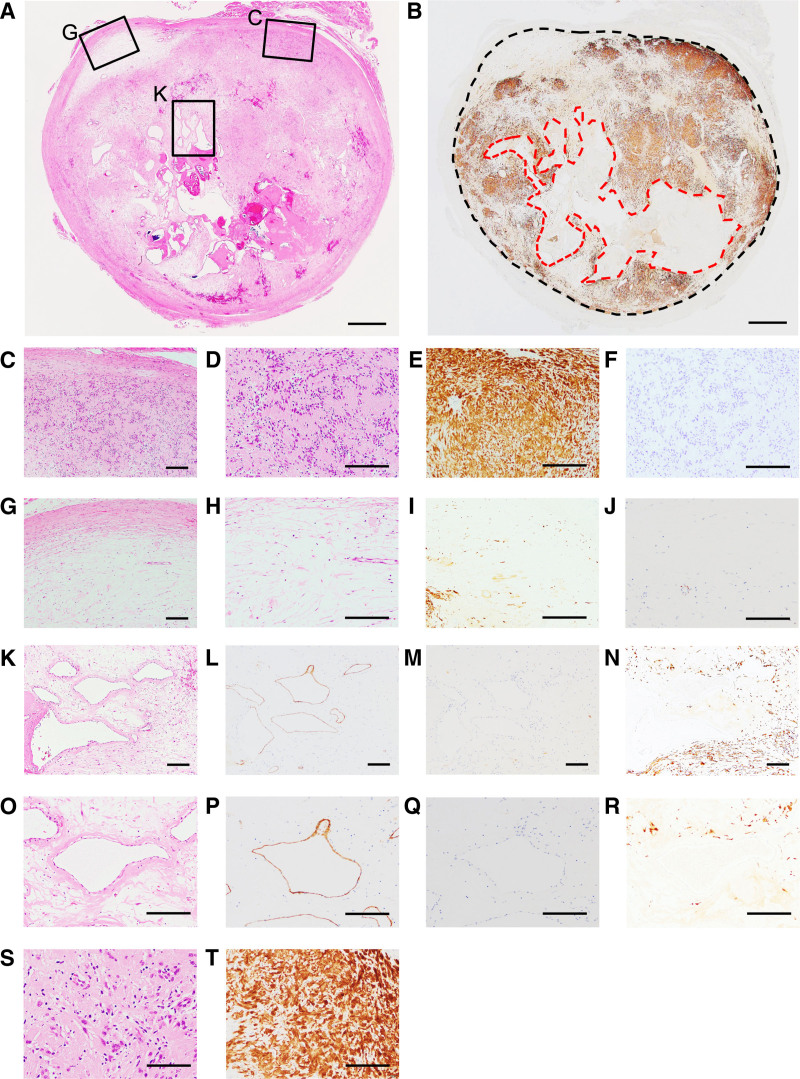
Histopathological and immunohistochemical features of the extirpated tumor mass. (A) A magnifying glass view showed the lesion encapsulated with thin fibrous tissue, and the lesion consisted of 2 major parts. Scale bar, 1 mm. Boxes show the typical histological features in the mass, and magnified images of the boxes in (A) are shown below. (B) The mass was composed of S100-positive/negative parts. Scale bar, 1 mm. The black dotted line indicates the mass margin. The red dotted line indicates the S100-negative area corresponding to the venous malformation-like areas. (C–J) Magnified images of the right and left square areas in (A) show proliferation of spindle cells in the cell-rich area and in the myxoid hypocellular area, indicating typical histological patterns of Antoni A (C–F) and Antoni B (G–J), respectively. More strongly magnified images of (C) and (G) are shown in (D–F) and (H–J), respectively. These spindle cells were positive for S100 (E, I), and negative for desmin (F, J). Scale bars, 100 μm. (K–N) Magnified images of the middle square area in (A) show vessels with hyalinized walls in the venous malformation-like areas. (O–R) More strongly magnified images of (K) are shown. The cells lining the vessel were positive for CD34 (L, P) and negative for D2-40 (M, Q). There was a certain distance between the ectatic cavernous vessels and the neoplastic S100-positive cells (N). S100-positive cells intervening between vessels were scarce (R). Scale bars, 100 μm. (S, T) More strongly magnified images in a part of (A) show several spindle-shaped to round cells with degenerative atypical nuclei. These spindle-shaped to round cells were positive for S100 (T). Scale bars, 50 μm.

A histopathological examination revealed that the lesion was encapsulated by fibrous connective tissue and consisted of 2 major parts with typical histopathological features (Fig. 3A). One lesion was composed of a proliferation of spindle cells in cell-rich and myxoid hypocellular areas, indicating typical histological features of Antoni A and Antoni B areas, as seen in schwannomas (Fig. 3A, C, D, G, H). Verocay bodies were also observed (Fig. 3D). Degenerative nuclear atypia was also observed in part (Fig. 3S, T). The other lesion showed several lobular foci of frequent ectatic, irregularly shaped and dilated vessels of varying sizes lined by attenuated endothelial cells without muscular support. The ectatic vessels were surrounded by thickened hyalinization. This lesion thus appeared to be a VM (Fig. 3A, K, L).

The following antibodies were used for immunohistochemical staining: Schwann cell marker S100 (1:2; cat. no. GA504; Agilent Technologies Inc., CA), muscular and myoepithelial markers alpha-SMA (1:500; cat. no. M0851; Agilent Technologies Inc.) and desmin (1:2; cat. no. IR606, Agilent Technologies Inc.), endothelial markers CD31 (1:200; cat. no. GA610; Agilent Technologies Inc.), and CD34 (1:10,000; cat. no. PA0212; Leica Biosystems, Wetzlar, Germany), lymphatic endothelial marker D2-40 (1:5; cat. no. 713451; Nichirei Biosciences Inc., Tokyo, Japan), and cell proliferation marker Ki-67 (1:100; cat. no. M 7240 clone MIB-1; Agilent Technologies Inc.). Immunohistochemically, spindle cells were positive for S100 (Fig. 3B, E, I, N, R, T) and negative for desmin (Fig. 3F, J). The attenuated endothelial cells were positive for CD31 (data not shown) and CD34 (Fig. 3L, P), and negative for D2-40 (Fig. 3M, Q). No invasion of S100-positive spindle cells was observed in the VM-like area. The demarcation between the schwannoma and the VM-like areas was clear (Fig. 3A, B). There was a certain distance between the ectatic cavernous vessels and neoplastic Schwann cells, which differed from the standard schwannoma (Fig. 3K, L, N, O, P, R). The schwannoma and VM-like areas were measured based on the S100-positive/negative areas as indicators using a BX53 system microscope (Olympus, Tokyo, Japan) and OLYMPUS cellSens standard 2.3 imaging software program (Olympus) and were 75.2% and 24.8%, respectively (Fig. 3B). Ki-67 positive cells were scattered, and the Ki-67 index of the spindle cells was approximately 3.0% (data not shown). Although a fresh hemorrhagic nest, possibly due to the needle biopsy, was observed from the center to the bottom of Figure 3A, cyst formation, calcification, and infiltration of siderophages/histiocytes intermingled with the neoplastic cells were not observed in the lesion. Based on these histopathological features and ancient changes,^[20–23]^ the tumor was diagnosed as an ancient schwannoma with VM-like features of the tongue rather than a composite lesion of VM within a schwannoma, because there were no definitive findings that would make these lesions a composite of independent lesions.

The symptoms of surgical insensitivity gradually improved. Neither local recurrence of the tumor nor limitation of tongue movement was observed during the 16-month follow-up period.

## 3. Discussion

In the present study, we report the case of a 29-year-old woman with an extremely rare ancient schwannoma with VM-like features of the tongue. To our knowledge, this is the first report of this type of ancient schwannoma on the tongue.

A composite lesion of VM within a schwannoma with histological findings similar to this case, located in the mediastinum, was first described as a rare entity by Willis.^[7]^ We found 24 cases of composite lesions of schwannomas coexisting with vascular malformations (Table 1).^[1,3,7–16]^ The male-to-female ratio was 1.4:1, and the patient’s age at diagnosis ranged from 17 to 77 years old, with an average age of 50.9 years old. The tumor size ranged from 2 to 10 cm, with an average size 4.1 cm. Thus, the present case was thus a relatively small mass (approximately 1 cm). Of these 24 cases, 11 cases occurred in the peripheral nerves other than the cranial nerves, as in the present case. Composite lesions of schwannoma and VM were observed in 22 of the 24 cases. As shown in Table 1, this type of lesion was the most prevalent among the reported composite lesions.^[2]^ This case revealed 2 intriguing features but was diagnosed as an ancient schwannoma with VM-like features based on the ancient changes^[20–23]^ because there were no definitive findings that would make these lesions a composite of independent lesions. However, there have been no reports of cases similar to this type that involve the tongue.

After the extirpation of schwannomas occurring in the head and neck region, a variety of postoperative symptoms may arise, such as auditory disturbance when these lesions occur in cranial nerve VIII, and pain, paresthesia, and swelling when they occur in the peripheral nerve, depending on the origin of the nerve.^[24]^ Therefore, it is important to make an accurate preoperative diagnosis to explain to the patient the potential risk of postoperative dysfunction in vascular malformation in addition to that in schwannomas in the treatment of composite lesions and ancient schwannomas with VM-like features. However, it is difficult to make an accurate preoperative diagnosis because schwannomas can be present at any site in the head and neck region and can mimic the clinical signs and symptoms of other lesions, such as benign tumors. A few intra-neural VMs of the peripheral nerves have also been reported.^[25]^ Therefore, an accurate preoperative diagnosis requires adequate investigation, including US, computed tomography, MRI, and biopsy.

An accurate differential diagnosis is important for management because it affects the treatment plan.^[26,27]^ Generally, in schwannomas of the tongue, US shows an elliptical mass with a well-defined border and a comparatively homogeneous echo texture with posterior echo enhancement.^[28]^ In contrast, with vascular malformations, US shows a well-defined mass and a net-like structure, and a hyperechoic area with a comet sign posterior acoustic shadow is observed in the lesion.^[28,29]^ On Doppler US, blood flow in the anechoic area is observed in vascular malformations of the tongue.^[28]^ In the present study, the interior of the lesion was heterogeneous with posterior acoustic enhancement. Doppler US revealed abundant blood flow at the center and margin of the mass. This finding differs from the typical findings in schwannomas. However, increased internal vascularization has also been reported on Doppler US in schwannomas.^[30]^ The target sign, which is a low signal in the center and a high signal in the periphery on the T2-weighted images, is a well-known characteristic of schwannomas.^[31]^ In another report, MRI of a schwannoma showed low intensity on T1-weighted images and high intensity on T2-weighted images,^[31]^ as observed in vascular malformations.^[32]^ However, this sign is not always visible.^[30,33]^ As a result, some cases may be clinically diagnosed as schwannomas, but histopathologically diagnosed as vascular malformations.^[20]^

In the present case, MRI showed that the intensity of the mass was low on T1-weighted images and high on T2-weighted water-only images. MRI revealed no obvious vascular structures inside the mass. Although schwannoma was suspected, it was difficult to rule out VMs or benign salivary gland tumors because of the abundant blood flow inside the mass on US, high signal intensity on T2-weighted water-only images, and high apparent diffusion coefficient. When differentiating between schwannomas and vascular malformations, it is difficult to make an accurate preoperative diagnosis using imaging alone. Therefore, careful evaluation and gathering of clinical data, including the presence of vascular structures, continuity with nerves and blood vessels, and target signs, are important for clinical diagnosis. In addition, as in the present case, the differential diagnosis should include the possibility of an ancient schwannoma.

A histopathological examination is very powerful for differentiating lesions from other lesions. Biopsies such as an incisional biopsy, fine-needle aspiration (FNA), and a core needle biopsy (CNB), are often required for a preoperative diagnosis. When incisional biopsy is used for the composite lesions, such as in 2 cases of arteriovenous malformation combined with schwannoma (Table 1), or for ancient schwannomas with VM-like features similar to the present case, there is a risk of bleeding.^[34,35]^ FNA and CNB are less burdensome to the patient but less diagnostically accurate than incisional biopsies because it is difficult to provide large tissue samples by FNA and CNB.^[35,36]^ However, for the tongue, CNB has a lower risk of bleeding than incisional biopsy.^[34,35]^ Based on a meta-analysis, CNB should be performed. When the diagnosis following CNB does not match the clinical presentation and radiographic findings, a surgical biopsy should be performed.^[35]^ In the present case, a small part of the tissue taken by CNB showed a schwannoma component in the lesion; however, VM-like features and ancient changes could not be detected. Therefore, it is necessary to confirm the overall histopathological findings of the extirpated sample to ensure an accurate and reliable diagnosis of this type of tumor.

Various hypotheses have been proposed to explain the tumorigenesis of composite lesions of concurrent VMs and schwannomas.^[2]^ However, such composite lesions must be interpreted with caution. In the present case, VM-like areas were located in the schwannoma surrounded by the tumor capsule membrane. This suggests that the schwannoma may have developed first, with a VM-like areas developing inside the tumor. In contrast, if a schwannoma develops around the cavernous malformation-like areas, the blood vessels of the VM-like areas will likely be flattened by the expansive development of the benign tumor. Notably, the hyaline-walled vessels of the VM-like areas in this case appeared to have been dilated in irregular shapes of varying sizes without compression or distortion due to the expansive development of the benign tumor (Fig. 3A, K, O). Thus, VM-like areas may develop concurrently with or after the current schwannoma. Based on these histopathological features and ancient changes,^[20–23]^ we made a diagnosis of ancient schwannoma with VM-like features of the tongue, because there were no definitive findings that would make these lesions a composite of independent lesions. Vascular malformation-like areas may be secondary changes and may be included in the histological findings of ancient schwannoma because several vessels of various sizes are seen within conventional schwannomas.^[20]^

To our knowledge, this is the first report of an ancient schwannoma with VM-like features of the tongue. This case is extremely rare because of the location and unique histological features of the lesion.^[20–23]^ Limited information on these composite lesions makes diagnosis difficult for clinicians. Awareness of the possibility of the rare tumors, as previously reported,^[17–19]^ can help clinicians to make an accurate preoperative diagnosis and plan appropriate treatment.

## Author contributions

**Conceptualization:** Tamotsu Kiyoshima.

**Data curation:** Tatsufumi Fujimoto, Kana Hasegawa, Wataru Kumamaru, Toru Chikui, Shinsuke Fujii, Kazunori Yoshiura, Shintaro Kawano, Tamotsu Kiyoshima.

**Funding acquisition:** Kana Hasegawa, Tamotsu Kiyoshima.

**Resources:** Tatsufumi Fujimoto, Wataru Kumamaru, Toru Chikui, Yuta Yanai, Minami Shibuya, Shinsuke Fujii, Kazunori Yoshiura, Shintaro Kawano, Tamotsu Kiyoshima.

**Supervision:** Kazunori Yoshiura, Shintaro Kawano, Tamotsu Kiyoshima.

**Writing – original draft:** Tatsufumi Fujimoto, Kana Hasegawa, Tamotsu Kiyoshima.

**Writing – review & editing:** Tatsufumi Fujimoto, Wataru Kumamaru, Toru Chikui, Yuta Yanai, Minami Shibuya, Shinsuke Fujii, Kazunori Yoshiura, Shintaro Kawano, Tamotsu Kiyoshima.
